# Global prevalence and antibiotic resistance profiles of carbapenem-resistant *Pseudomonas aeruginosa* reported from 2014 to 2024: a systematic review and meta-analysis

**DOI:** 10.3389/fmicb.2025.1599070

**Published:** 2025-07-21

**Authors:** Tsepo Ramatla, Jane Nkhebenyane, Kgaugelo E. Lekota, Oriel Thekisoe, Maropeng Monyama, Conrad Chibunna Achilonu, George Khasapane

**Affiliations:** ^1^Centre for Applied Food Safety and Biotechnology, Department of Life Sciences, Central University of Technology, Bloemfontein, South Africa; ^2^Unit for Environmental Sciences and Management, North-West University, Potchefstroom, South Africa; ^3^Department of Life and Consumer Sciences, University of South Africa, Johannesburg, South Africa; ^4^Thoracic Diseases Research Unit, Departments of Medicine and Biochemistry, Mayo Clinic College of Medicine, Rochester, MN, United States

**Keywords:** pooled prevalence estimate, CRPA, antibiotic resistance, global, meta-analysis

## Abstract

**Introduction:**

Carbapenem-resistant *Pseudomonas aeruginosa* (CRPA) represents a global threat, but the global distribution of carbapenem resistant bacteria remains a critical issue in public health.

**Methods:**

We conducted a systematic review and meta-analysis on the global pooled prevalence estimate (PPE) of CRPA and their antibiotic resistance. The systematic review protocol was registered with PROSPERO (CRD42024579654). This study was carried out following the preferred reporting items for systematic reviews and meta-analyses (PRISMA) guidelines. Heterogeneity between studies was assessed using Cochrane Q test and I^2^ test statistics based on the random effects model. Comprehensive meta-analysis software v4.0 was used to analyze the pooled prevalence of CRPA.

**Results:**

A total of 163 studies (both clinical and screening samples) containing a total of 58,344 cases from 39 countries were included in this study. The overall PPE of CRPA was 34.7% (95% CI: 0.316–0.37.8) for both clinical and screening samples. Meropenem had a PPE of 31.2% (95% CI: 0.272–0.352) and imipenem had the lowest PPE of 27.7% (95% CI: 0.238–0.319). Japan had the highest PPE at 98.2% (95% CI: 0.482–0.100) of CRPA, and the lowest was observed for Saudi Arabia at 13.9% (95% CI: 0. 064–0. 277). CRPA is widespread on five continents except Australia and Antarctica, while the highest PPE is in Europe at 47.6% (95% CI: 0.359–0.595) and the lowest in Asia at 32, 8% (95% CI: 0.293–0.364). The relatively higher PPE of CRPA was observed in Europe during the year interval 2014–2017 at 95.4% (95% CI: 0.388–0.999), followed by Africa from the year 2022–2024 with 38.5% (95% CI: 0.243–0.550). Ceftazidime was significantly higher in studies conducted before 2019 with a PPE of 44.7% (95% CI: 0.246% – 0.668), while CRPA after 2019 had a higher resistance to cefoperazone/sulbactam with a PPE of 17.3% (95% CI: 0.050–0.455).

**Discussion:**

This review indicates that the prevalence of CRPA is generally high and varies significantly between countries. To prevent the emergence of CRPA and antibiotic resistance, future initiatives should prioritise strengthening laboratory capacity for early detection of antibiotic resistance.

## 1 Introduction

*Pseudomonas aeruginosa* is a Gram-negative, rod-shaped bacterium that belongs to the *Pseudomonadaceae* family (Marchaim et al., 2012; Abdalhamid et al., 2016). It is an opportunistic human pathogen that can cause a variety of infections in the blood, skin, respiratory tract, and urinary tract (Golle et al., 2017; Narimisa et al., 2024) and is common for healthcare-associated infections (HAIs) (Büchler et al., 2023). Antimicrobial resistance (AMR) is one of the greatest threats to public health worldwide, leading to significant increases in morbidity, mortality, and treatment failure for microbial infections, as well as economic losses to individuals and nations. The primary mechanisms by which resistance is mediated in Gram-negative bacteria are *ampC*-lactamases, carbapenemases, and extended spectrum lactamases (ESBL) (Schill et al., 2017; Marouf et al., 2023). Carbapenems are effective drugs against bacterial pathogens and resistance to them is considered a major threat to public health, especially among notorious nosocomial pathogens such as *P. aeruginosa* (Arowolo et al., 2023). Carbapenemases are categorized into three main classes: Class A (*Klebsiella pneumoniae* carbapenemase, KPC), Class B (metallo-*β*-lactamases, including NDM, VIM, and IMP), and Class D (oxacillinases, such as OXA-48-like carbapenemases) (Sheu et al., 2019).

The management of infections caused by this bacterium has become increasingly challenging due to the continued rise in antibiotic resistance and, more recently, the emergence of multidrug-resistant strains (Odoi et al., 2021; Deshwal et al., 2023). Carbapenems are used to treat infections caused by multidrug-resistant Gram-negative bacteria, but the selective pressure imposed by their use has contributed to the emergence of carbapenem-resistant strains (Rai et al., 2014; Sheu et al., 2019; Abubakar et al., 2022). Several studies reported antibiotic resistance of *P. aeruginosa*, including AMR against carbapenem (Çopur et al., 2021; Souza et al., 2021; Canton et al., 2022; Saha et al., 2022; Męcik et al., 2024; Pappa et al., 2024; Shahab et al., 2024; Yano et al., 2024). Treatment options can be severely restricted when bacteria develop resistance to carbapenem antibiotics, which are generally reserved for the treatment of multidrug-resistant bacterial infections (Deshwal et al., 2023).

Several systematic reviews of carbapenem-resistant CRPA worldwide have been published including study on carbapenem resistance in *Acinetobacter baumannii* and *P. aeruginosa* in sub-Saharan Africa, Carbapenemase-producing non-glucose-fermenting Gram-negative Bacilli in Africa, *P. aeruginosa* and *Acinetobacter baumannii* (Kindu et al., 2020), outbreak investigations after identifying CRPA (Büchler et al., 2023), and prevalence of meropenem-resistant *P. aeruginosa* in Ethiopia (Gobezie et al., 2024). Though there is limited information on comprehensive data available to estimate the global prevalence of CRPA and their antibiotic resistance. This systematic review and meta-analysis aimed to provide a comprehensive overview of the global prevalence of CRPA and their antibiotic resistance patterns, based on peer-reviewed published global data.

## 2 Materials and methods

### 2.1 Protocol registration

This study was registered in the International Prospective Register of Systematic Reviews (PROSPERO) with registration no: CRD42024579654.

### 2.2 Systematic review protocol

The Preferred Reporting Items for Systematic Reviews and Meta-analyses (PRISMA) guidelines were used in the data extraction, screening, and analysis process, which have been confirmed on a checklist (Supplementary Table S1). This included searching database systems for potentially relevant articles, assessing their suitability for review and determining their relevance.

### 2.3 Search strategy

This study used several database systems, including Web of Science (https://lnkd.in/gAdNu8iz/ 03-04April/2024), Scopus (https://www.scopus.com/ 06/April/2024) Google Scholar (https://scholar.google.com/ 08-16/April/2024) ScienceDirect (https://lnkd.in/gBrgWFQ2/ 20-22/April/2024) PubMed (https://lnkd.in/gABzu4AD/ 22-25/April/2024) and EMBASE (https://www.embase.com/ 01-02/April/2024). The search strategy was not limited by language. Literature searches were conducted from 2014 to May, 2, 2024, using keywords comprising of “epidemiology,” “carbapenem,” “meropenem,” “imipenem,” “antibiotics resistant,” “*Pseudomonas*,” “multidrug resistance,” “VIM,” “IMP,” carbapenem-resistant *Pseudomonas aeruginosa*, “CRPA,” antibiotic resistance, “nosocomial,” “infections,” “health care related,” meropenem resistant, “antibiotics,” “antimicrobials,” “hospital setting.” Certain “MeSH” terms were used to find articles relevant to the study. The search strategy is detailed in Supplementary Table S2. The last search took place on 02 May 2024. No attempt was made to obtain further information or retrieve unpublished studies from the authors of the original manuscripts.

### 2.4 Study selection

The three authors TR, GK, and JN assessed the suitability of journal titles and abstracts for the inclusion and exclusion criteria. Two reviewers independently examined each study found through the search, focusing on the title, abstract, and selected full text. A third reviewer clarified any discrepancies. Chapter books, reviews, and conference abstracts were excluded. The full text of only English-language journal articles was incorporated. We screened titles and abstracts, then retrieved and downloaded relevant full-text articles via library resources and online databases. Full-text reviews of journal articles examining carbapenem-resistant *P. aeruginosa* and their resistance to antibiotics were selected.

### 2.5 Inclusion and exclusion criteria

Criteria for inclusion of studies were: (i) studies investigating the prevalence of carbapenem-resistant *P. aeruginosa* and their antibiotic resistance in humans. The following exclusion criteria were used, (ii) no total number of isolates, (iii) no abstracts, reviews, experiments, thesis, preprints and book chapters, and (iv) an unclear number of carbapenem-resistant *P. aeruginosa* and their antibiotic resistance.

### 2.6 Data extraction

To determine eligibility, full versions of potentially relevant articles were obtained by two authors independently (TR and GK) from the final selected studies. Data from each article were independently compiled and entered into a spreadsheet, including author names, publication year, location, total number of isolates, and total number of samples collected, which were entered into a Microsoft Excel for further analysis. Text, tables, and figures were used to extract data.

### 2.7 Quality appraisal

To confirm the methodological soundness of the research articles selected for quantitative synthesis, two authors independently used the Joanna Briggs Institute (JBI) Critical Appraisal Tools Checklist 2017 review guideline for prevalence studies (Ramatla et al., 2022). Studies that achieved a score of five or higher for the evaluation criteria were included.

### 2.8 Meta-analysis

Only journal articles that specifically addressed the antibiotic resistance of CRPA were included in the meta-analysis. We performed the meta-analysis using the Comprehensive Meta-Analysis (CMA) program Software v.4.0.^1^ The random effects model, equipped with 95% confidence intervals (CI), produced pooled estimates (PP) by using the reciprocal of the sample variance and a constant variable across the population effects to weigh each study. To assess Cochran heterogeneity (Q) within studies and percentage variation in prevalence, Higgins I^2^ (inverse variance) and the Cochran Q method were used. One can define low, moderate, or high heterogeneity as I^2^ values of less than 25, 50%, and more than 75%, respectively, while values close to 0% indicate no heterogeneity. A random effects model was used to create all pooled estimates. Statistical significance was determined by considering heterogeneity with a *p*-value less than 0.05 (*p* < 0.05). The *p*-values correspond to the heterogeneities between studies from a Chi-squared test of the null hypothesis that there is no heterogeneity. A subgroup analysis of study results was performed based on carbapenem, meropenem, imipenem, antibiotic resistance, countries, continents, published year, and carbapenem-resistant *P. aeruginosa,* and their antibiotic resistance. As part of the meta-analysis, subgroup analyzes that contained studies with fewer than three studies were excluded.

### 2.9 Publication bias

Publication bias was determined using an inverted funnel plot, Egger’s and Begg’s bias indicator tests, and the visual eye test. The Begg-Mazumdar bias indicator test was used to study the impact of publishing and selection bias.

## 3 Results

### 3.1 Search and screening results

**Figure 1:**
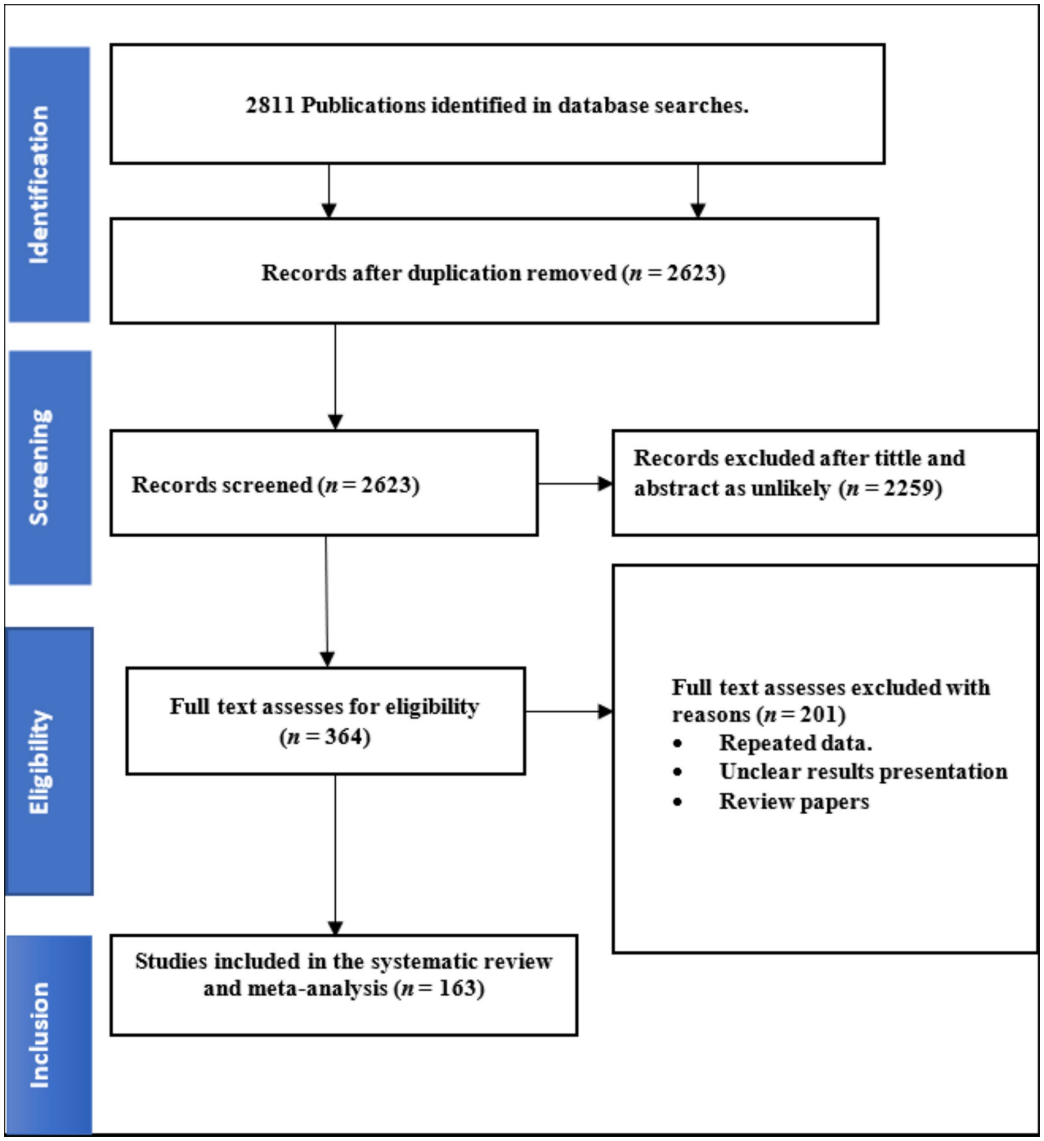
The PRISMA diagram shows the study selection process.

A total of 2,811 studies were retrieved after the initial search was conducted across four databases (Scopus, PubMed, ScienceDirect, EMBASE, Google Scholar and Web of Science), subsequently removing 188 duplicate articles. After removing duplicates and reviewing study titles and abstracts, 2,623 articles were excluded from further consideration. A total of 364 studies were initially considered eligible and were thus subjected to full text evaluation; thereafter, 163 studies were eligible for inclusion (Figure 1). The Joanna Briggs Institute (JBI) Critical Review Quality Assessment score ranges from 1 to 9. All 163 studies included in our analysis received a score of five or higher.

### 3.2 Characteristics of eligible studies

All included studies were published from 2014 to 2024, with most studies conducted between 2021 and 2024. Table 1 shows the annual distribution of published articles from all continents. Of the five continents, Asia had the highest number of published studies (*n* = 95), followed by Europe (*n* = 19), South America (*n* = 16), Africa (*n* = 15), and very few came from North America (*n* = 13). A total of 5 (3.06%) studies were conducted from more than one country/continent. The number of CRPA isolates confirmed worldwide ranged from 1 to 9,750 isolates. Numerous studies have examined the expression of antibiotic resistance genes for the carbapenem class, including *bla*_IVIM_ 45 (25.6%), *bla*_IIMP_ 22 (12.5%), *bla*_INDM_ 18 (10.2%), *bla*_IKPC_ 17 (9.7%), *bla*_ISPM_ 7 (9.7%), *bla*_oxa48_ 7 (9.7%), *bla*_ISPM1_ 5 (2.8%), *bla*_ISIM_ 4 (2.3%), and *bla*_IGIM_ 4(2.3%). Most CRPA isolates showed resistance to amikacin (n = 28; 15.9%), ceftazidime (n = 26; 14.8%), gentamicin (*n* = 25; 14.2%), cefepime (no = 25; 1.2%), ciprofloxacin (no = 24;1.2%) and Piperacillin-tazobactam (no = 20; 11.4%).

**Table 1:** Annual distribution of published articles from seven different continents.

Year	Asia	Africa	Europe	North America	South America	Australia	Antarctica	Total
2014	3	0	0	0	3	0	0	6
2015	2	2	1	0	1	0	0	6
2016	2	1	0	2	4	0	0	9
2017	3	0	4	2	2	0	0	11
2018	8	0	2	0	0	0	0	10
2019	10	2	1	1	0	0	0	14
2020	12	3	1	0	0	0	0	16
2021	13	3	2	3	1	0	0	22
2022	11	1	3	1	1	0	0	18
2023	22	2	4	2	2	0	0	34
2024	8	4	1	1	3	0	0	12

### 3.3 Meta-analysis results on overall prevalence

The global PPE of CRPA, as well as a summary of the subgroup analysis, are shown in Table 2. A total of 291,715 isolates were confirmed as CRPA, while 19,858 were confirmed to be CRPA multiple drug resistant (CRPA-MDR) isolates. Studies examining the prevalence of CRPA and their antibiotics in humans have found high heterogeneity based on methods, countries, continents, years and antibiotic resistance profiles (Tables 2, 3). The PPE of CRPA in the present study was 34.7% (0.347; 95% CI: 0.316–0.378, I^2^ = 99.2%) from 163 studies. Among studies reporting meropenem resistance, the PPE was 27.7% (0.277; 95% CI: 0.238–0.319, I^2^ = 98.7%), while imipenem had the least PPE of 31.2% (0.312; 95% CI: 0.272–0.352, I^2^ = 99.2%).

**Table 2:** Subgroup analysis of carbapenem-resistant *Pseudomonas aeruginosa* reported from 2014 to 2024.

Pooled prevalence estimates					Measure of heterogeneity			Publication bias
Risk factors	No. studies	No. of Isolates/patient	No. of positives Isolates/patient	Prevalence 95% CI (%)	*Q*	*I* ^2^	*Q*–*P*	P-value
Carbapenem								
Carbapenem	169	303,620	53,619	34.7% (31.6–37.8)	20222.009	99.164	*p* < 0.0001	0.487
Meropenem	94	120,897	18,038	27.7% (23.8–31.9)	7238.195	98.701	*p* < 0.0001	0.415
Imipenem	90	114,309	23,565	31.2% (27.2–35.2)	5274.596	98.294	*p* < 0.0001	0.402
Methods								
MIC	57	69,732	23,588	39.3% (32.4–46.7)	10127.247	99.447	*p* < 0.0001	0.091
Disk Diffusion	51	66,923	12,359	44.1% (37.2–51.3)	2219.833	97.748	*p* < 0.0001	0.470
VITEK®2	20	79,729	10,920	38.0% (23.9–54.4)	2056.715	99.076	*p* < 0.0001	0.948
BD Phoenix	7	6,566	2,137	21.4% (13.1–33.0)		97.027	*p* < 0.0001	0.050
E-test	8	715	444	55.2% (36.5–72.6)	111.964	93.748	*p* < 0.0001	0.620
Mortality								
Mortality	22	10,930	6,552	6.9% (2.3–19.2)	5564.783	99.641	*p* < 0.0001	0.204
Countries								
China	44	92,362	15,324	24.4% (19.5–30.1)	4863.343	99.116	*p* < 0.0001	0.943
USA	12	109,513	19,370	32.8% (19.3–49.9)	11859.540	99.907	*p* < 0.0001	0.680
Brazil	12	2,574	656	45.3% (23.6–69.0)	552.946	98.011	*p* < 0.0001	0.583
Saudi Arabia	4	2,547	278	13.9% (6.4–27.7)	102.502	97.073	*p* < 0.0001	0.496
Iran	17	2,415	1,161	70.7% (53.1–83.7)	581.629	97.249	*p* < 0.0001	0.364
Colombia	3	1,008	233	24.9% (1.5–87.6)	327.693	99.390	*p* < 0.0001	0.601
India	8	5,875	329	32.2% (11.1–64.5)	574.411	98.781	*p* < 0.0001	0.804
Germany	4	615	148	31.6% (6.9–74.1)	138.919	97.840	*p* < 0.0001	0.496
Italy	4	5,514	1,186	20.7% (12.8–31.9)	62.806	95.223	*p* < 0.0001	0.496
Korea	3	251	186	95.3% (27.6–99.9)	23.540	91.504	*p* < 0.0001	0.301
Egypt	5	439	130	27.3% (15.5–43.3)	24.802	83.872	*p* < 0.0001	0.327
Japan	4	1972	1948	98.2% (48.2–100)	45.628	93.425	*p* < 0.0001	0.500
Greece	3	313	129	79.6% (9.9–99.3)	95.094	97.897	*p* < 0.0001	0.601
Continents								
Asia	97	185,720	33,704	32.8% (29.3–36.4)	7921.644	98.788	*p* < 0.0001	0.526
Africa	15	1,673	538	38.5% (24.3–55.0)	257.483	94.563	*p* < 0.0001	0.053
Europe	19	7,991	2,298	47.6% (35.9–59.5)	601.232	96.840	*p* < 0.0001	0.242
South America	16	3,723	958	40.9% (21.2–64.1)	881.939	98.413	*p* < 0.0001	0.804
North America	13	109,430	19,380	33.3% (20.1–49.8)	11865.296	99.899	*p* < 0.0001	0.714
Years								
2013–2020	83	262,622	45,492	34.1% (29.9–38.7)	18149.351	99.548	*p* < 0.0001	0.032
2021–2024	89	55,348	14,948	36.7% (31.2–42.6)	8158.143	98.921	*p* < 0.0001	0.222

**Table 3:** Pooled prevalence estimates and 95% CI of antibiotic resistance profiles of carbapenem-resistant *Pseudomonas aeruginosa.*

Pooled prevalence estimates					Measure of heterogeneity			Publication bias
Risk factors	No. of studies	No. of isolates/patient	No. of positives isolates/patient	Prevalence 95% CI (%)	*Q*	*I* ^2^	*Q*–*P*	*p*-value
Antibiotics								
Amikacin	28	11,768	768	31.1% (17.5–49.1)	1428.292	98.110	*p* < 0.0001	0.323
Ceftazidime	26	11,459	934	51.1% (35.6–66.4)	911.450	97.257	*p* < 0.0001	0.440
Cefepime	25	11,814	1,044	44.5% (31.2–58.7)	722.937	96.680	*p* < 0.0001	0.743
Ciprofloxacin	24	1772	936	54.5% (43.6–64.9)	311.021	92.605	*p* < 0.0001	0.619
Piperacillin-tazobactam	20	11,372	703	32.5% (17.0–53.1)	1361.551	98.605	*p* < 0.0001	0.299
Piperacillin	11	715	324	45.9% (30.9–61.7)	128.252	92.203	*p* < 0.0001	0.815
Polymyxin B	4	10,259	18	1.0% (0.1–8.0)	52.745	94.312	*p* < 0.0001	0.496
Colistin	6	10,149	40	3.0% (0.6–13.5)	97.328	94.863	*p* < 0.0001	0.347
Gentamicin	25	11,825	893	36.2% (26.2–47.5)	493.804	95.140	*p* < 0.0001	0.815
Aztreonam	17	10,980	617	54.4% (31.3–75.8)	952.096	98.319	*p* < 0.0001	0.322
Levofloxacin	14	1,301	614	48.0% (35.7–60.5)	187.505	93.067	*p* < 0.0001	0.869
Cefoperazone/Sulbactam	4	374	200	53.0% (39.4–66.2)	16.016	81.269	*p* < 0.0001	0.500
MDR	28	16,510	20,210	24.3% (19.4–30.0)	3624.019	99.034	*p* < 0.0001	0.688

#### 3.3.1 Prevalence of carbapenem resistant *Pseudomonas aeruginosa*

The prevalence data in subgroups classified by the countries showed that studies from Japan registered the highest PPE of CRPA at 98.2% (0. 982; 95% CI: 0.482–0.100, Tau = 15.511, I^2^ = 93.4%), while Saudi Arabia had the lowest at 13.9% (0. 139; 95% CI: 0. 064–0. 277, Tau = 1.644, I^2^ = 91.1%) (Table 2). However, other countries were not included in the meta-analysis due to the small number of studies. Based on the continental distribution, which included only five of the seven continents, the highest prevalence of CRPA was reported in Europe with a PPE of 47.6% (0.476; 95% CI: 0.359–0.595, Tau = 0.779, I^2^ = 96.8%), followed by South America with a PPE of 40.9% (0.409; 95% CI: 0.212–0.641, Tau = 1.801, I2 = 98.4%) (Table 2). The CRPA PPE was below 40% on three continents: Africa 38.5% (0.385; 95% CI: 0.243–0.550, Tau = 0.656, I^2^ = 94.5%), North America 33.3% (0.333; 95% CI: 0.201–0.498, Tau = 0.777, I^2^ = 99.8%) and Asia 32.8% (0.328; 95% CI: 0.293–0.364, Tau = 1.016, I^2^ = 98.7%). Furthermore, a relatively higher PPE of CRPA was observed in Europe during the year interval 2014–2017 at 95.4% (0.954; 95% CI: 0.388–0.999, Tau = 14.677, I^2^ = 97.6%), followed by Africa from the year 2022–2024 with 87.5% (0.875; 95% CI: 0.449–0.636, Tau = 1.593, I^2^ = 89.6%) while Asia had a PPE of 54.4% (0.544; 95% CI: 0.449–0.636, Tau = 1.370, I2 = 97.6%) in 2018–2021(Table 2).

When it came to tests for antibiotic resistance, the E-test had the highest PPE at 55.2% (0.552; 95% CI: 0.65–0.726, Tau = 1.001, I^2^ = 93.7%), followed by MIC with a PPE of 39.3% (0.393; 95% CI: 0.324–0.46.7, Tau = 1.167, I^2^ = 99.4%), disk diffusion with a PPE of 44.1% (0.441; 95% CI: 0.372–0.513, Tau = 0.904, I^2^ = 97.7%), VITEK®2 with a PPE of 38.0% (0.380; 95% CI: 0. 239–0.544, Tau = 1.440, I^2^ = 99.1%), and lastly, PPE of 21.4% (0.214; 95% CI: 0.131–0.330, Tau = 0.487, I^2^ = 97.0%) for BD Phoenix.

#### 3.3.2 Subgroup analysis by carbapenem-resistant *Pseudomonas aeruginosa*

We analyzed the antibiotic resistance of CRPA and 34 of the 163 studies reported resistance of carbapenem-resistant *P. aeruginosa* (Table 3). A subgroup study of antibiotic resistance found that the highest PPE of antibiotic resistance was against ciprofloxacin with 54.5% (0.545; 95% CI: 0.436–0.649, I^2^ = 92.6%) followed by aztreonam 54.4% (0.544; 95% CI: 0.313–758, I^2^ = 98.3%), cefoperazone/sulbactam 53.0% (0.530; 95% CI: 0.394–662, I^2^ = 81.3%), ceftazidime 51.1% (0.511; 95% CI: 0.356–0.664, I^2^ = 97.3%), levofloxacin 48.0% (0.480; 95% CI: 0.357–0.605, I^2^ = 93.1%), piperacillin 45.9% (0.459; 95% CI: 0.309–0.617, I^2^ = 92.2%), cefepime 44.5% (0.445; 95% CI: 0.312–0.587, I^2^ = 96.7%), gentamicin 36.2% (0.632; 95% CI; 0.262–0.475, I^2^ = 95.1%), piperacillin-tazobactam 32.5% (0.325; 95% CI; 0.170–0.531, I^2^ = 98.6%), amikacin 31.1% (0.311; 95% CI; 0.175–0.491, I^2^ = 98.1%), colistin 3.0% (0.003; 95% CI; 0.003–0.135, I^2^ = 94.9%), polymyxin B 1.0% (0.010; 95% CI; 0.0.001–0.080, I^2^ = 94.3%). The CRPA resistance rates in different continents are described in detail in Supplementary Table S3.

We conducted a meta-analysis of these antibiotics and compared antimicrobial resistance rates for CRPA before and after 2019. The rate of CRPA antimicrobial resistance to ceftazidime with PPE was 44.7% (0.447; 95% CI, 0.246–0.668%, I^2^ = 95.5) and was significantly higher in studies conducted before 2019, followed by ciprofloxacin with PPE at 41.2% (0.412; 95% CI, 0.256–0.588, I^2^ = 94.8). However, after 2019, CRPA showed significantly higher resistance to cefoperazone/sulbactam with a PPE of 17.3% (0.173; 95% CI, 0.050–0.455, I^2^ = 96.2) (Supplementary Table S4).

#### 3.3.3 Subgroup analysis by carbapenem-resistant *Pseudomonas aeruginosa*-multidrug resistance

**Figure 2:**
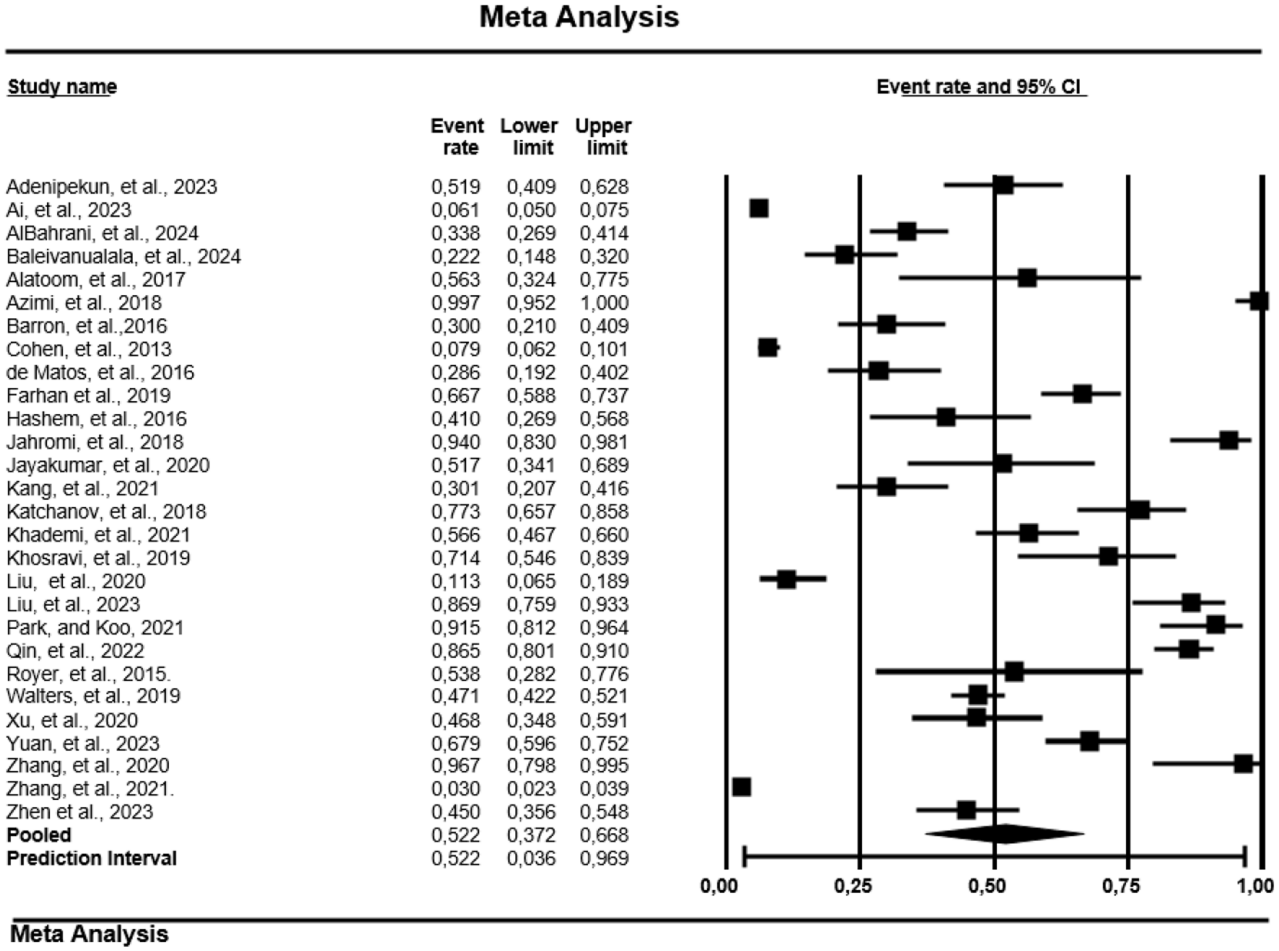
Forest plot showing the pooled estimates of multidrug resistance of carbapenem-resistant-*P. aeruginosa* globally. The diamond at the base indicates the pooled estimates from the overall studies.

The study also analyzes the MDR of 28 of the 163 papers reporting on CRPA (Figure 2). Africa recorded the highest PPE at 63.2% (0.632; 95% CI; 0.211–0.917, Tau = 3.168, I^2^ = 96.9%). Followed by Asia at 28.4% (0.284; 95% CI; 0.151–0.469, Tau = 2.709, I^2^ = 98.9%) and finally North America with PPE of 17.7% (0.177; 95% CI; 0.106–0.280, Tau = 0.350, I^2^ = 99.7%). However, South America and Europe were not included in the analysis due to the small number of studies.

#### 3.3.4 Subgroup analysis by carbapenem-resistant *Pseudomonas aeruginosa* genes

The PPE of antibiotic resistance genes for CRPA (Table 4) considered in this meta-analysis were as follows: *bla*_GIM_ with high PPE of 22.3% (0.223; 95% CI: 0.056–0.583, I^2^ = 94.2%), *bla*_SPM_ 21.9% (0.219; 95% CI: 0.067–0.525, I^2^ = 94.3%), *bla*_VIM_ 21.3% (0.213; 95% CI: 0.144–0.302, I^2^ = 96.6%), *bla*_SPM1_ 19.9% (0.199; 95% CI: 0.088–0.390, I^2^ = 83.5%), *bla*_SIM_ 15.9% (0.159; 95% CI: 0.041–0.455, I^2^ = 62.5%), *bla*_IMP_ 14.2% (0.142; 95% CI: 0.078–0.243, I^2^ = 95.2%), *bla*_NDM_ 8.4% (0.084; 95% CI: 0.038–0.177, I^2^ = 94.7%), and *bla*_KPC_ 7.5% (0.075; 95% CI: 0.049–0.114, I^2^ = 88.5%). However, due to a low number of studies, genes for cefametazole, ceftriaxone, tobramycin, minocycline, ertapenem, carbenicillin, doripenem, tobramycin, and cefotaxime were not included in the analysis.

**Table 4:** Pooled prevalence rate and 95% CI of antibiotic resistance genes of carbapenem-resistant *P. aeruginosa* based on meta-analysis.

Resistant genes	Number of studies	Number of Isolates	Prevalence % (95%CI)	*I^2^* (%)	Begg and Mazumdar rank *p*-value
*bla*_VIM_	45	1,035	21.3 (14.4–30.2)	96.622	0.945
*bla*_IMP_	22	330	14.2 (7.8–24.3)	95.227	0.259
*bla*_NDM_	18	164	8.4 (3.8–17.7)	94.688	0.676
*bla*_KPC_	17	280	7.5 (4.9–11.4)	88.454	0.284
*bla*_SPM_	7	88	21.9 (6.7–52.5)	94.302	0.881
*bla*_oxa48_	7	79	19.7 (9.3–36.8)	88.435	0.652
*bla*_SPM1_	5	45	19.9 (8.8–39.0)	83.495	0.500
*bla*_SIM_	4	45	15.9 (4.1–45.5)	92.469	0.500
*bla*_GIM_	4	52	22.3 (5.6–58.3)	94.248	0.496

#### 3.3.5 Prevalence of carbapenem-resistant *Pseudomonas aeruginosa* mortality

**Figure 3:**
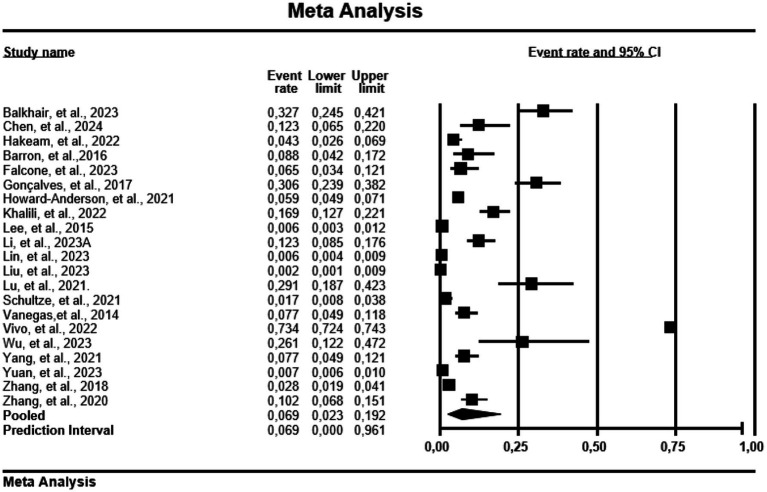
Forest plot showing mortality prevalence in carbapenem resistance-*P. aeruginosa*. The diamond at the base indicates the pooled estimates from the overall studies.

Regarding mortality, China recorded a PPE of 3.3% (0.033; 95% CI: 0.009–0.110, Tau = 3.400, I^2^ = 98.1%) in 153 patients with CRPA (Figure 3). However, countries like USA, Italy, Brazil, Omen, and Iran were not included in analysis due to low number of studies.

### 3.4 Risk of publication bias of included studies

The Begg and Mazumdar rank correlation test, Egger regression test, and funnel plots were additionally used to measure publication bias and check for symmetry. We examined publication bias using the funnel plot. The funnel plots of the estimates suggested publication bias (Supplementary Figure S1) for Asia, the year interval 2018–2021 (*p* = 0.025).

## 4 Discussion

In this systematic review and meta-analysis, we conducted an in-depth study to investigate the prevalence of carbapenem-resistant *P. aeruginosa* and its antibiotic resistance reported worldwide between 2014 and 2024, using over 163 peer reviewed published papers. Thus, we conducted a subgroup analysis of CRPA and their antibiotic resistance. Several articles that only reported on the prevalence of *P. aeruginosa* and not carbapenem-resistant strains were excluded from our systematic literature review. However, the aim of this study was to include only studies reporting on CRPA and their antibiotic resistance profiles.

Carbapenems are important broad-spectrum antimicrobials of last resort, therefore, resistance to them means increased infections, mortalities, length of hospital stays, and treatment costs (Arowolo et al., 2023). In this context, the results of our meta-analysis and systematic review of the prevalence of CRPA worldwide provide important new information about the terrain of antibiotic resistance. This study recorded an overall PPE of 34.7% for CRPA, with Asia, Africa, Europe, South America and North America having a PPE of 32.8, 38.5, 47.6, 40.9, 33.3 and 47.8%, respectively. This observation of prevalence is higher than that reported in sub-Saharan Africa in 2022 (8%), and the prevalence in the African continent of 21.36% (Kindu et al., 2020). Countries vary in the amount of antibiotics they consume, which may explain why different countries have different prevalence rates of resistant strains.

The current study revealed that the PPE for imipenem and meropenem is 31.2 and 27.7%, respectively. These results are comparable to the prevalence of 30% meropenem resistance reported in Turkey (Acar et al., 2019). Another study from Latin America shows a susceptibility rate of 57% to meropenem and 52% to imipenem among *P. aeruginosa* isolates (Labarca et al., 2016). Due to the increasing use of carbapenems to treat ESBL-producing infections, the number of carbapenem-resistant Enterobacteriaceae is increasing worldwide (Ramatla et al., 2023).

Studies used in this analysis show that the *bla*_GIM_, *bla*_SPM_, *bla*_VIM_, and *bla*_GIM-1_ genes are the most frequently occurring carbapenem-resistant genes in CRPA, with a PPE of 22.3, 21.9, 21.3, and 19.7%, respectively. The studies conducted in sub-Saharan Africa, China, and India reported the *bla*_VIM_ and *bla*_IMP_ genes as the most common carbapenemase genes detected in *P. aeruginosa* isolates (Verma et al., 2019; Wang et al., 2021; Arowolo et al., 2023). Carbapenem-resistant *P. aeruginosa* isolates have been associated with a variety of carbapenemases, including *bla*_IMP_, *bla*_VIM_, *bla*_KPC_, *bla*_GES_, *bla*_NDM_, *bla*_GIM_, and *bla*_SPM_ (McCracken et al., 2019; Wang et al., 2021). Verona integron–encoded metallo-*β*-lactamase (*bla*_VIM_) is the most identified CRPA worldwide (Diene and Rolain, 2014). It is also the most common carbapenemase identified in *P. aeruginosa* in some countries like the United States (Kracalik et al., 2022). The metallo-*β*-lactamase *bla*_GIM-1_ (imipenemase) has so far only been found in clinical isolates of *P. aeruginosa* (Rieber et al., 2012). The *bla*_KPC_, *bla*_NDM_, and *bla*_IMP_ exhibited a low prevalence. Class D oxacillinases (OXA)-type enzymes such as OXA- 48-like is one of the major classes of carbapenemases (Sheu et al., 2019). A number of OXA-carbapenemases, including the OXA-48-like carbapenemases, have proliferated within Enterobacterales and have emerged as a key mechanism for carbapenem resistance in these isolates across multiple countries (Poirel et al., 2012; Evans and Amyes, 2014; Ferous et al., 2024). Although data on the transfer dynamics of antimicrobial resistance genes (ARGs) between clinical environments are limited, there have been several instances where the same ARG was simultaneously identified on hospital surfaces and in patients (Andersson and Hughes, 2017; Lerminiaux and Cameron, 2019).

In this systematic review and meta-analysis, we also report the presence of CRPA and their antibiotic resistance. Using a random-effect model, the pooled prevalence of ciprofloxacin had the highest PPE of 54.5%. The literature reveals instances of ciprofloxacin-resistant *P. aeruginosa* (CRPAs) in chronic ear infections called suppurative otitis (Jang and Park, 2004; Jang et al., 2009), from patients diagnosed with *P. aeruginosa* nosocomial infection (Qin et al., 2022). There is a possibility that ciprofloxacin-resistant organisms first appeared in the human domain, since this antibiotic is frequently utilized in human healthcare (Ramatla et al., 2022). The AR against aztreonam had the second highest PPE of 54.4%. Aztreonam plays a crucial role in treating infections caused by metallo-*β*-lactamase (MBL)-producing carbapenem-resistant Enterobacterales and carbapenem-resistant CRPA (Ding et al., 2023). Therefore, the CRPA resistance to aztreonam is a concern for public health. The emergence of *P. aeruginosa* strains with multidrug resistance (MDR) is due to their possession of enormous genetic alterations that can develop a variety of factors associated with resistance to various antibiotics (Al-Abedi and Al-Mayahi, 2019). This study observed the proportion of public health impact of multidrug-resistant isolates (MDR) from 28 studies. We believe our results should generate hypotheses and stimulate additional research into the treatment of patients with CRPA.

Furthermore, the current study calculated the PPE for mortality, which is 8.6% from 14 studies. This prevalence is lower as compared to those reported before our study, ranging from 21.0 to 68.3% in Spain, Brazil, the USA, and China (Peña et al., 2012; Dantas et al., 2014; Buehrle et al., 2017; Shi et al., 2019; Vivo et al., 2022; Zhen et al., 2023). Therefore, our study supports the notion that patient mortality can be attributed to CRPA which is a very significant issue.

The subgroup analysis at the continental level showed that the PPE was higher in Europe (47.6%) and South America (40.9%). This is a higher prevalence than 15% reported in Ethiopia by Gobezie et al. (2024) for CRPA. We observed an increase in CRPA of 16.7 to 38.5% in Africa between 2018 and 2021 and 2022–2024, of 95.4 to 54.4% in Asia between 2014 and 2017 and 2018–2021 and around 19.6 to 50.9% in North America between 2018 and 2021 2021 and in between 2022 and 2024. These periodic increases suggest that there could be a global increase in CRPA infections, or the increased number of research funds could be a possible reason for this change.

Additionally, this study found that 56.4% of studies were conducted in Asia, compared to other continents such as Europe at 11.1%, South America (9.3%), Africa (8.7%) and North America (7.6%). No data were available from Australia/Oceania. Our findings were consistent with a previous study reporting that Asian countries have a high prevalence of *P. aeruginosa* due to chronic wound infections globally (Phan et al., 2023). The differences could be explained by the availability of research funding or the high number of *P. aeruginosa* infections in Asian countries compared to other continents. Furthermore, countries vary in the amount of antibiotics they consume, which may explain why different countries experience different prevalence rates of resistant strains.

## 5 Limitations

There are few limitations to our systematic review and meta-analysis: (a) The search strategy was limited to articles published in English, meaning that there may have been articles published in other languages that were missed. (b) The findings of some countries may not be accurate since the number of studies was limited. (c) Compared to other countries, some had more research reports than others. (d) There were no studies reported in Australia/Oceania continent. (e) No data were available in some countries. (f) When there is a high level of heterogeneity, it is difficult to evaluate the actual results of statistically significant publication bias tests. (g) The number of studies from some countries were unusually high, which may have influenced the overall estimate. (h) The pooled prevalence of some countries and continents was not calculated because there are few published studies. (i) Data from large scale surveillance systems was not included. (j) There may have been articles published in other languages that were missed since the search strategy was limited to articles published in English. (k) Nevertheless, meta-analyses that comprise fewer than ten studies or show high heterogeneity among studies might produce misleading outcomes from these evaluation tools. When heterogeneity is high, assessing the true results of statistically significant publication bias tests becomes very difficult. Given the significant variability among analyses, readers ought to be careful when analyzing pooled analyses and subgroups. (l) This study only examined mortality data from China, as other countries had insufficient studies to be included.

## 6 Conclusion

Our comprehensive meta-analysis provides important updated global prevalence of CRPA from 2014 to 2024, which appears to be increasing or spreading. There is an urgent demand for comprehensive surveillance studies, involving both hospitals and communities worldwide, to accurately measure the global prevalence of these multidrug-resistant pathogens. This study presents robust and valuable data that can serve as a useful reference for clinicians and researchers in informing them about the CRPA. Globally, alternative treatments for CRPA should be well researched, planned, and implemented.

## Data Availability

The original contributions presented in the study are included in the article/Supplementary material, further inquiries can be directed to the corresponding author.

## Footnotes

^1^ https://www.meta-analysis.com/
